# Loneliness Is Associated With Decreased Support and Increased Strain Given in Social Relationships

**DOI:** 10.1111/psyp.70105

**Published:** 2025-07-10

**Authors:** Emily M. Kent, Anita Restrepo, Kelly E. Faig, Sabina Raja, Stephanie J. Dimitroff, Karen E. Smith, Greg J. Norman

**Affiliations:** ^1^ Department of Psychology University of Chicago Chicago Illinois USA; ^2^ Department of Psychology Hamilton College Clinton New York USA; ^3^ Department of Psychology University of Montana Missoula Montana USA; ^4^ Department of Psychology Rutgers University – Newark Newark New Jersey USA

**Keywords:** loneliness, parasympathetic, strain, support

## Abstract

Prolonged loneliness can be detrimental to both mental and physical health. However, variability in how individuals respond to loneliness can shape health outcomes. Here, we explored whether loneliness is related to perceptions of support and strain given in family and friend relationships. Specifically, we assessed whether resting high‐frequency heart rate variability (HF‐HRV), a measure of parasympathetic nervous system activity that has been linked to emotion regulation and flexible adaptation, moderates self‐evaluation of support and strain given. Participants from the Midlife Development in the US (MIDUS) dataset who had measures of loneliness, perceived support given, and perceived strain given in relationships, and resting HF‐HRV were included in the current study. Loneliness was associated with decreased support and increased strain given in both family and friend relationships. Resting HF‐HRV showed a trend‐level moderation of the association between loneliness and perceived strain given to family, with the relationship being stronger for individuals with lower resting HF‐HRV. No moderation was observed for strain given to friends. These results indicate that loneliness is linked to more negative self‐evaluations of social support and strain, and that resting HF‐HRV may partly buffer these effects in family relationships.

## Abstract 2

Our findings add a new perspective on self‐evaluation of social contributions as a mechanism underlying differences in loneliness adults in mid‐life. We provide evidence that negative self‐evaluations of support and strain given in social relationships are associated with the perception of social isolation. Specifically, we find associations with perceived strain given to family and resting parasympathetic activity, which may have implications for the development of prolonged loneliness and associated long‐term health risks.

## 1 Introduction

Loneliness, or perceived social isolation, is associated with long‐term health risks, including heightened susceptibility to depression, cardiovascular disease, and increased mortality (Ohman 2008; Cacioppo et al. 2011; Holt‐Lunstad et al. 2015; Jaremka et al. 2013; Rico‐Uribe et al. 2018). However, individuals vary in their cognitive and behavioral responses to loneliness in ways that can influence these long‐term impacts (Hawkley and Cacioppo 2010; Smith and Pollak 2022; Vanhalst et al. 2018). Identifying what drives variability in responses can provide insight into who is most at risk of long‐term health impacts and how to intervene. One underexplored factor that may contribute to long‐term health outcomes is shifts in perceptions of one's own role and contribution within social networks. These self‐perceptions likely shape when and how individuals socially engage, resolving or exacerbating loneliness. This study aims to address this gap by exploring how feelings of loneliness relate to self‐assessments of providing social support and strain and what drives variability in those assessments. Specifically, we examined whether flexible and adaptive affective and behavioral regulation, as indexed by resting high‐frequency heart rate variability (HF‐HRV), a measure of parasympathetic cardiac control (Quigley et al. 2024; Thayer and Lane 2000), influences these dynamics.

For social species, strong social connections are critical for adaptive functioning, offering benefits such as resource access and protection from environmental threats (Alexander 1974). Loneliness is defined as the perception of deficits in the quality or quantity of social relationships (Perlman and Peplau 1981). It signals a potential loss of those social relationships and serves as a warning to motivate repair or maintenance of social connections (Cacioppo and Hawkley 2009). As such, loneliness is thought to heighten sensitivity to potential threats, particularly social threats, simultaneously facilitating repair or maintenance of bonds in safe contexts and avoidance of further rejection in unsafe ones (Cacioppo et al. 2011; Qualter et al. 2015; Spithoven et al. 2017). Some evidence does suggest that loneliness induces shifts toward enhanced processing of social information, particularly cues that may indicate social threat, including increased sensitivity to negative emotions like sadness and fear (Vanhalst et al. 2015), better recall of negative social information (Gardner et al. 2005; Hess and Pickett 2010), increased visual attention toward social threat (Bangee et al. 2014), and increased expectations of social rejection (Jones et al. 1981; but see Lodder et al. 2016; Okruszek et al. 2021 for alternative perspectives). While this heightened sensitivity to social information, and negative social information in particular, can facilitate reconnection in safe social situations and avoidance of further rejection in unsafe ones, individuals vary in their ability to effectively leverage these two motivations flexibly (Smith and Pollak 2022). For some, this heightened sensitivity to negative information results in hypervigilance and social withdrawal, creating a feedback loop that prolongs loneliness and potentially exacerbates health risks (Cacioppo and Hawkley 2009).

One manifestation of loneliness‐related vigilance is shifts in perceptions of the social world, including persistent attitudes and beliefs about oneself and others. Lonely individuals report more negative evaluations of both themselves and others (Jones et al. 1983; Spithoven et al. 2017), including reduced trustworthiness, prosocial behaviors, support, attractiveness, and social desirability (Bellucci 2020; Heinrich and Gullone 2006). In ambiguous social situations, particularly those involving potential exclusion, lonely individuals are more likely to attribute hostile intent to others, reinforcing perceptions of social threat (Qualter et al. 2013; Restrepo et al. 2024). Notably, these negative evaluations appear to intensify in closer relationships, suggesting that the context of the relationship plays a significant role in shaping lonely individuals' perceptions (Tsai and Reis 2009). Together, these findings indicate that loneliness increases sensitivity to social threats through the perpetuation of negative social cognitions, which can ultimately undermine social connection and promote the continuation of loneliness.

While research has focused on how loneliness impacts perceptions of others, the specific ways in which loneliness shapes self‐perception of social capacity are less clear. Loneliness‐related negative evaluations are not limited to perceptions of others; they extend to individuals' assessments of their own capacity to engage in social relationships. Loneliness has been consistently related to lower self‐perceived social skills, social competency, self‐regard, and feelings of inferiority (Lodder et al. 2016; Spithoven et al. 2017). Perceptions of low social competence, in turn, can result in a self‐fulfilling prophecy where the perception of an inferior ability to engage in effective social interactions results in reduced quality of social interactions (Leary 1999; Bierman and McCauley 2010; Lee et al. 2010), contributing to a perpetuation of loneliness. Despite evidence that loneliness shapes self‐evaluations related to social competence with downstream effects, more work is needed to bridge our understanding of how specific aspects of self‐perception relate to social behavior.

Two factors that may impact the way lonely individuals use social perceptions to inform social motivations and subsequent behavior are the amount of support and strain they perceive themselves to provide in social relationships (Lodder et al. 2016). Social support, defined as the resources and/or attention available, depends upon an individual registering the support and interpreting it as such (Shumaker and Brownell 1984; Uchino et al. 2022). Typically, social support is viewed as being received by the individual from others; however, individuals can also provide support within their relationships. Research indicates that lower levels of loneliness are often associated with individuals who actively provide social support to close members of their network (De Jong Gierveld et al. 2009; Rodrigues et al. 2014). Moreover, the perception of being unable to offer support mediates the relationship between loneliness and psychological distress (Bentley et al. 2022). Conversely, social strain results from the perception of negative experiences in social interactions, such as conflict, instability, and repeated negative interactions (Newsom et al. 2005). Recent studies have demonstrated that loneliness is linked to the perception of being a burden to others (Del Sequeros et al. 2021). This self‐perception of being a burden can exacerbate feelings of isolation and reinforce the cycle of loneliness. If individuals believe themselves to add negatively to their social groups, they may be less likely to engage in behaviors that facilitate social connection (e.g., initiating conversations), thereby perpetuating social avoidance (Bédard et al. 2014).

The relationship between loneliness and self‐perceptions of providing support or causing strain likely varies by both individual and relationship type. For example, De Jong Gierveld and Dykstra (2008) found that loneliness was linked to support given differently in parent, sibling, and child relationships: providing support to siblings was associated with lower loneliness, whereas providing support for children corresponded with increased loneliness. This suggests a complex interplay between loneliness and perceptions of social contributions, with evidence indicating that the effects of giving support vary based on relational context.

In addition to the role of relational context, the effects of perceived support and strain may also differ as a function of individual capacity for behavioral and affective regulation. One factor that could provide insight is resting high‐frequency heart rate variability (HF‐HRV) which indexes cardiac parasympathetic nervous system (PNS) activity. The PNS is intricately connected to higher order neural structures, such as the ventromedial prefrontal cortex (vmPFC), which modulate self‐regulatory processes like emotion regulation and social engagement through inhibitory control (Thayer and Koenig 2025; Porges 2007; Smith et al. 2017). Inhibition from the PNS and associated neural regions permits adaptive responses to situational demands. Resting HF‐HRV indexes this inhibitory activity and, as such, has been identified as a physiological marker of flexible and effective emotion regulation. Individuals with higher resting HF‐HRV demonstrate greater capacity to adjust adaptively to context, including behaviors that foster social connection (Isgett et al. 2017; Smith et al. 2020). Higher resting HF‐HRV is associated with increased social interaction, cooperation, and perceptions of social support in the environment (Beffara et al. 2016; Geisler et al. 2013; Kemp et al. 2017). Conversely, lower resting HF‐HRV is linked to avoidance behaviors and poor emotional regulation in social contexts (Balzarotti et al. 2017; Katahira et al. 2014) as well as heightened threat vigilance. Effective regulation of emotions and behavior not only involves high levels of resting inhibition but also necessitates efficient withdrawal of inhibition in response to threats or cognitive demands (Thayer and Koenig 2025; Porges 2007).

Inducing loneliness has been shown to decrease the withdrawal of cardiac PNS inhibition (measured with HF‐HRV) during social information processing (Piejka et al. 2021), suggesting that loneliness itself may impair an individual's physiological capacity for social engagement. Additional work has shown a negative relationship between loneliness and vagal flexibility—a combination measure indexing both higher resting HF‐HRV and greater task‐related withdrawal (Muhtadie et al. 2015). In the context of the present study, it is possible that individuals with lower resting HF‐HRV may perceive their behaviors as less supportive and as contributing to relational strain. This is consistent with work suggesting that HF‐HRV serves as a potential marker for the underlying processes that influence social behaviors and related loneliness perceptions (Isgett et al. 2017; Smith et al. 2020), which can reinforce further isolation. Indeed, resting HF‐HRV has been found to moderate individuals' tendency to approach or withdraw in response to loneliness (Smith and Pollak 2022), and lower resting HF‐HRV has been associated with perceptions of burdensomeness and unmet interpersonal needs (MacNeil et al. 2023). Together, this suggests that the PNS may index an individual's propensity for adaptive participation or contribution to social relationships.

Here, we assessed whether loneliness is related to perceptions of support and strain given in family and friend relationships. We expected that loneliness would predict less perceived support and more perceived strain given to both friends and family. We also considered whether resting HF‐HRV moderates the relationships between loneliness and individual perceptions of support and strain given. We expected stronger associations between loneliness and perceptions of support and strain for individuals with lower levels of resting HF‐HRV. If increased resting HF‐HRV is linked to reduced negative perceptions of social contribution in individuals experiencing loneliness, this suggests that resting HF‐HRV may index an aspect of emotional or behavioral flexibility that allows individuals to buffer some of the adverse effects of feeling lonely by promoting more adaptive responses.

## 2 Method

### 2.1 Data

Data were obtained from the Midlife Development in the United States (MIDUS) Series. All measures in the current study come from the MIDUS Refresher Biomarker study (2012–2016) excluding demographic variables, which were obtained from the MIDUS Refresher study Survey (2011–2014) and the Milwaukee African American Sample (2012–2013). Biomarker data and psychosocial constructs were collected during 2‐day clinic visits. All participants provided informed consent, and all study procedures were approved by the Institutional Review Board at the University of Wisconsin‐Madison. Secondary analysis was approved by the Institutional Review Board at the University of Chicago.

Participants were included in the analysis if they participated in the MIDUS Refresher Biomarker study (2012–2016) and had measures of resting HF‐HRV, loneliness, and perceived support and strain in relationships. The Biomarker study included a national sample of US adults as well as a subsample of African American adults from Milwaukee, Wisconsin. Participants were excluded if their baseline ECG waveform quality was labeled as Missing Data, Period Not Run, or Inapplicable (*N* = 39). See Table 1 for the demographic characteristics of the sample.

**TABLE 1:** Demographic and descriptive characteristics of participants (*N* = 824).

Variable	*N*	%
Age	Mean = 50.42 (SD = 13.33)	
Loneliness	Mean = 12.64 (SD = 4.45)	
HF‐HRV at rest (ms^2^)	Mean = 5.12 (SD = 1.36)	
Household income	Mean = $91,079.59 (SD = $66,322.07)	
**Gender**		
Men	390	47.33
Women	434	52.67
**Race**		
White	580	70.39
Black	153	18.57
Native American	20	2.43
Asian	12	1.46
Other	54	6.55
No response	5	0.61
**Married**		
Yes	536	65.05
No	286	34.71
No response	2	0.24
**Regular smoking**		
No	220	26.7
Yes	89	10.8
No response	515	62.5
**Heart condition**		
No	301	36.53
Yes	514	62.38
No response	9	1.09
**Number of medications used**		
Zero	413	50.12
One	172	20.87
Two	144	17.48
Three	74	8.98
Four	19	2.31
Five	2	0.24

### 2.2 Measures

#### 2.2.1 Loneliness

A modified 7‐item version of the UCLA Loneliness Scale‐Version 3 (Russell 1996) was used to assess feelings of loneliness in participants. Questions are rated on a four‐point scale (Never, Rarely, Sometimes, and Often). Sample items include “I feel isolated from others” and “People are around me but not with me.” Higher total scores indicate higher levels of loneliness. This questionnaire had high internal consistency in the current sample (alpha = 0.87). Scores ranged from 7 to 28 (*M* = 12.54, SD = 4.45).

#### 2.2.2 Support Given

Support given to family and friends was assessed with the Support Given to Friends and Support Given to Family scales provided in the MIDUS Refresher Biomarker study (2012–2016). These scales were developed based on studies by Schuster et al. (1990) and Walen and Lachman (2000) and have previously been used to assess perceptions of social contribution (Fitzgerald and Morgan 2023; Srirangarajan et al. 2020; Kane and Krizan 2021). Questions are rated on a four‐point Likert scale. Support given to friends was assessed with four items. Total scores were calculated for each scale by taking an average of the individual item scores. Higher total scores indicate higher levels of support given. Support given to family was assessed with two items. Participants were instructed to report on support given to family “not including spouse or partner.” Support given to family had moderate internal consistency in the current sample (alpha = 0.70), and scores ranged from 1 to 4 (*M* = 3.72, SD = 0.51). Similarly, support given to friends had moderate internal consistency in the current sample (alpha = 0.72), and scores ranged from 1 to 4 (*M* = 3.63, SD = 0.40). Consistencies in both scales align with prior research (Fitzgerald and Morgan 2023; Srirangarajan et al. 2020; Kane and Krizan 2021). All scale items can be found in Table S1.

#### 2.2.3 Strain Given

Strain given to family and friends was assessed with Strain Given to Friends and Strain Given to Family scales. These scales were developed based on studies by Schuster et al. (1990) and Walen and Lachman (2000). They have previously been used to assess perceptions of social contributions (Fitzgerald and Morgan 2023). These two scales each have four items and are rated on a four‐point scale (A lot, Some, Rarely, and Not at All). For the Strain Given to Family Scale participants were instructed to report on strain given to family “not including spouse or partner.” Higher total scores indicate higher levels of strain given. Strain given to family had moderate internal consistency in the current sample (alpha = 0.63), and scores ranged from 1 to 4 (*M* = 1.76, SD = 0.50). Similarly, strain given to friends had moderate internal consistency in the current sample (alpha = 0.64), and scores ranged from 1 to 4 (*M* = 1.58, SD = 0.45). Consistencies in both scales align with prior research (Fitzgerald and Morgan 2023; Srirangarajan et al. 2020; Kane and Krizan 2021). See Supporting Information for a complete list of scale items (Table S1).

#### 2.2.4 Resting High‐Frequency Heart Rate Variability (HF‐HRV)

During the psychophysiological protocol from the MIDUS Refresher Biomarker Study (2012–2016), participant baseline measures for physiological outcomes were measured at rest. Baseline measures of parasympathetic cardiac control were derived from an electrocardiogram (high frequency—0.15–0.50 Hz—heart rate variability, reported as the natural logarithm of power in ms^2^). As reported in the MIDUS protocol, HF‐HRV was derived using a Fast Fourier Transform (FFT) methods, following those described by Deboer et al. (1984). HF‐HRV, an index of vagally mediated rhythmic fluctuations in heart rate, is a pure measure of parasympathetic cardiac control (Quigley et al. 2024) The electrocardiogram (ECG) was collected using a standard lead II configuration and digitized at a sampling rate of 500 Hz by a 16‐bit National Instruments analog‐to‐digital (A/D) board installed in a microcomputer. Baseline measurements were collected in a 10‐min baseline. MIDUS research staff visually inspected all ECG waveforms and scored data in 60‐s epochs. Subjects were excluded from analysis for technical issues or missing data.

In accordance with current guidelines (Quigley et al. 2024), HF‐HRV was used as the primary measure of parasympathetic activity. However, to ensure robustness, we conducted parallel analyses using the root mean square of successive differences (RMSSD). These analyses yielded consistent results, which are reported in the Supporting Information.

### 2.3 Data Analytic Strategy

The relationships between loneliness, resting parasympathetic activity, and support and strain given within social relationships were assessed using Ordinary Least Squares (OLS) multiple regression. Loneliness and parasympathetic activity were included as predictors of strain and support given to family and friends in four separate regression models. All variables were standardized (*M* = 0, SD = 1) prior to analysis so that coefficients represent estimates of effect sizes (Lorah 2018). Moderation effects were assessed using an interaction term. To probe interactions, the emmeans (Lenth 2022) and sjPlot (Lüdecke 2021) packages were used to run simple slopes analyses. RStudio (R version 4.3.3; RStudio Team 2020) was used to fit all models. A post hoc power analysis was conducted using the effect sizes obtained in the model. This analysis indicated a sample of 200 is sufficient to obtain these effects with 80% power. Our current sample size of *n* = 824 exceeds this threshold, suggesting there is sufficient power to detect current effects. To address the issue of multiple testing and reduce the risk of Type I error, we report both unadjusted *p* values and false discovery rate (FDR)‐adjusted *p* values for regression analyses. All analysis code is available on OSF (https://osf.io/kxgqs).

Following prior established guidelines for analysis of HF‐HRV in the MIDUS sample by Knight et al. (2020), age, gender, and medical conditions or medications which could influence resting PNS activity were included as covariates. Additional factors, such as smoking status, socioeconomic status, and other psychological factors, which may influence HF‐HRV and loneliness but were not central to the primary question, were included in a series of exploratory models in Tables S2 and S3. Given recent findings that gender moderates the association between heart rate variability (HRV) and social perception (Piejka et al. 2024), we also ran an exploratory analysis to examine a three‐way interaction between gender, loneliness, and HF‐HRV, which is included in the supplemental materials.

## 3 Results

### 3.1 Main Effects of Support and Strain

As hypothesized, loneliness was associated with perceived support and strain in friend and family relationships. Higher levels of loneliness were associated with decreased perceived support given to friends (*β* = −0.34, SE = 0.04, *p* < 0.001, FDR‐adjusted *p* < 0.001, 95% CI [−0.41, −0.27]) and family (*β* = −0.30, SE = 0.04, *p* < 0.001, FDR‐adjusted *p* < 0.001, 95% CI [−0.37, −0.23]). Further, increased levels of loneliness predicted increased perceived strain given in friend (*β* = 0.17, SE = 0.04, *p* < 0.001, FDR‐adjusted *p* < 0.001, 95% CI [0.10, 0.24]) and family (*β* = 0.20, SE = 0.04, *p* < 0.001, FDR‐adjusted *p* < 0.001, 95% CI [0.13, 0.27]) relationships.

### 3.2 Interactions With Resting HF‐HRV

**FIGURE 1:**
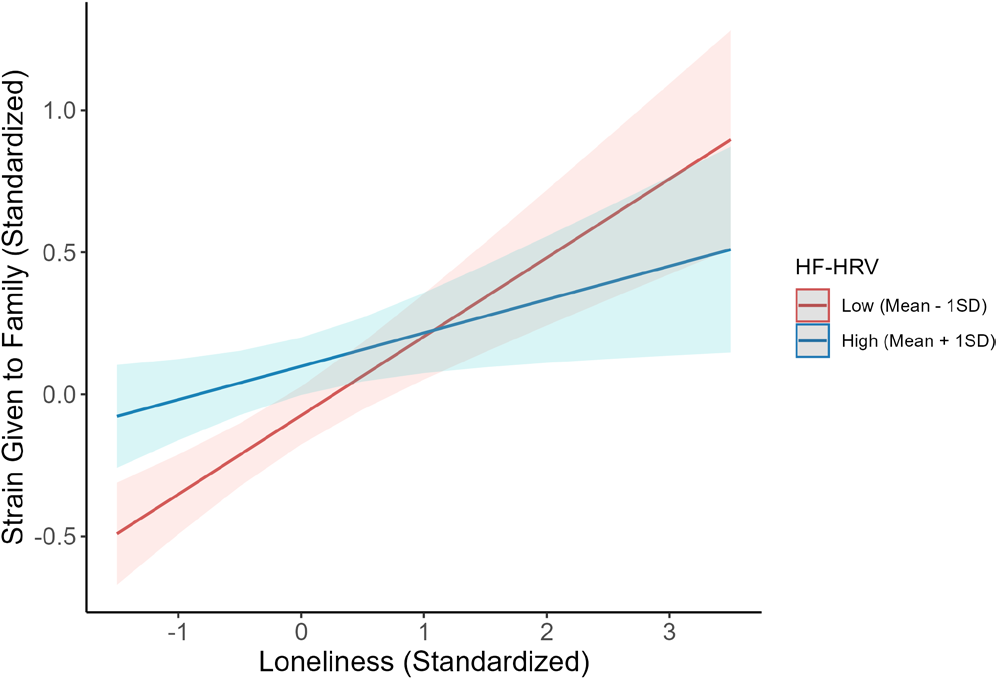
The positive relationship between loneliness and strain is more pronounced for individuals with lower resting HF‐HRV. Loneliness predicted strain given to family (*β* = 0.28, SE = 0.05, *p* < 0.001, 95% CI [0.17, 0.38]) at lower HF‐HRV levels, but the relationship was attenuated (*β* = 0.12, SE = 0.05, *p* = 0.039, 95% CI [0.02, 0.22]) for higher HF‐HRV levels. Higher and lower resting HF‐HRV are represented as one standard deviation from the mean for visualization (continuous measure used in analyses). Graph shows fitted values based on model predictions for the unadjusted model. Error bars reflect 95% confidence intervals.

There was evidence in support of our hypothesis that resting HF‐HRV would moderate self‐evaluations, but only for perceptions of strain. Resting HF‐HRV interacted with loneliness to predict strain given to family (*β* = −0.08, SE = 0.04, *p* = 0.030, FDR‐adjusted *p* = 0.051, 95% CI [−0.15, −0.008]), such that the effect of loneliness on strain was most pronounced for individuals with lower resting HF‐HRV (Figure 1). A simple slopes analysis at one standard deviation above and below the mean for resting HF‐HRV was conducted to probe the interaction effect. At higher HF‐HRV levels (one SD above the mean), loneliness was significantly associated with perception of strain given to family (*β* = 0.12, SE = 0.05, *p* = 0.021, 95% CI [0.02, 0.22]). At lower resting HF‐HRV levels (one SD below the mean), the relationship between loneliness and strain given to family was also significant and had a steeper slope (*β* = 0.28, SE = 0.05, *p* < 0.001, 95% CI [0.17, 0.38]). This result suggests that the relationship between loneliness and strain given to family is more pronounced for individuals with lower resting HF‐HRV. The relationship between resting HF‐HRV and strain given to family becomes a trending effect after the inclusion of covariates (*β* = −0.07, SE = 0.04, *p* = 0.062, FDR‐adjusted *p* = 0.124, 95% CI [−0.14, 0.003]). Resting HF‐HRV did not interact with loneliness to predict strain given to friends (*β* = −0.03, SE = 0.04, *p* = 0.432, FDR‐adjusted *p* = 0.519, 95% CI [−0.10, 0.04]), support given to friends (*β* = 0.04, SE = 0.04, *p* = 0.277, FDR‐adjusted *p* = 0.369, 95% CI [−0.03, 0.11]), or support given to family (*β* = 0.02, SE = 0.04, *p* = 0.626, FDR‐adjusted *p* = 0.683, 95% CI [−0.05, 0.09]) (Tables 2 and 3).

**TABLE 2:** Regression results showing associations between loneliness, baseline HF‐HRV, and their interaction with supportive and strained behaviors toward friends and family.

	Model 1: Support given to friends, *β* (SE)	Model 2: Support given to family, *β* (SE)	Model 3: Strain given to friends, *β* (SE)	Model 4: Strain given to family, *β* (SE)
Loneliness	−0.338**	−0.297**	0.171**	0.198**
	(0.035)	(0.036)	(0.036)	(0.037)
Baseline HF‐HRV	0.103**	0.014	0.051	0.087*
	(0.035)	(0.036)	(0.036)	(0.037)
Loneliness:Baseline HF‐HRV	0.038	0.018	−0.029	−0.080
	(0.035)	(0.036)	(0.036)	(0.037)
Constant	−0.011	−0.006	−0.005	0.012
	(0.035)	(0.036)	(0.036)	(0.036)
Observations	735	735	732	732
*R* ^2^	0.118	0.086	0.033	0.052
Adjusted *R* ^2^	0.115	0.082	0.029	0.048
Residual SE	0.952 (df = 731)	0.968 (df = 731)	0.981 (df = 728)	0.986 (df = 728)
*F* statistic	32.694** (df = 3; 731)	22.814** (df = 3; 731)	8.401** (df = 3; 728)	13.395** (df = 3; 728)

*Note:* Linear regression models without covariates (i.e., unadjusted for other variables) predicting support and strain given to friends and family from loneliness, baseline high‐frequency heart rate variability (HF‐HRV), and their interaction. Standardized beta coefficients (*β*) and standard errors (SE) are reported. All *p* values are corrected for false discovery rate (FDR).

* *p* < 0.05.

** *p* < 0.01.

**TABLE 3:** Adjusted regression models examining the associations between loneliness, baseline HF‐HRV, and their interaction with supportive and strained behaviors toward friends and family.

	Model 1: Support given to friends, *β* (SE)	Model 2: Support given to family, *β* (SE)	Model 3: Strain given to friends, *β* (SE)	Model 4: Strain given to family, *β* (SE)
Loneliness	−0.320**	−0.292**	0.150**	0.186**
	(0.036)	(0.037)	(0.037)	(0.038)
Baseline HF‐HRV	0.077	0.003	0.029	0.029
	(0.038)	(0.040)	(0.040)	(0.040)
Gender	0.428**	0.093	−0.101	0.108
	(0.071)	(0.074)	(0.074)	(0.075)
Age	0.028	0.014	−0.134**	−0.145**
	(0.041)	(0.043)	(0.043)	(0.043)
Medical conditions	−0.061	0.011	0.092	0.033
	(0.083)	(0.086)	(0.087)	(0.088)
Medications	0.004	−0.052	0.050	0.009
	(0.042)	(0.044)	(0.044)	(0.044)
Loneliness:Baseline HF‐HRV	0.042	0.021	−0.016	−0.069
	(0.035)	(0.036)	(0.037)	(0.037)
Constant	−0.209**	−0.063	−0.010	−0.074
	(0.071)	(0.074)	(0.075)	(0.075)
Observations	728	728	725	725
*R* ^2^	0.161	0.088	0.050	0.070
Adjusted *R* ^2^	0.153	0.079	0.040	0.061
Residual SE	0.934 (df = 720)	0.972 (df = 720)	0.977 (df = 717)	0.983 (df = 717)
*F* statistic	19.804**(df = 7; 720)	9.949** (df = 7; 720)	5.340** (df = 7; 717)	7.736** (df = 7; 717)

*Note:* Models include covariates identified in Knight et al. (2020): age, gender, medical conditions, and medications. Standardized beta coefficients (*β*) and standard errors (SE) are reported. All *p* values are corrected for false discovery rate (FDR).

** *p* < 0.01.

## 4 Discussion

In the current study, we assessed the relationship between loneliness and individual perceptions of support and strain given within family and friend relationships. We found that higher levels of loneliness were associated with the perception of less support and more strain given across all types of relationships. We also examined whether resting HF‐HRV moderated differences in perceived support and strain given. We found trend‐level evidence that resting PNS activity moderates the relationship between loneliness and the amount of strain individuals reported giving to family members. The positive association between loneliness and strain was stronger for individuals with lower resting HF‐HRV. However, resting HF‐HRV did not moderate the effect of loneliness for support given to either friends or family. Together, these findings suggest that loneliness is associated with more negative self‐evaluations of social relationships. Further, those with lower resting HF‐HRV may be more susceptible to viewing their own contributions negatively, especially within family relationships.

Our findings enhance the understanding of how loneliness affects social self‐evaluations by demonstrating that loneliness is associated with more negative self‐assessments. Specifically, we identify two key factors—perceptions of support and strain given to family and friends that help explain how lonely individuals interpret social information that may influence their behavior. Loneliness has previously been linked to negative self‐evaluations (Chen and Graham 2012; Spithoven et al. 2017) and less confidence in social competency (Lodder et al. 2016; Vanhalst et al. 2013). Prior work has also shown that loneliness is related to strain in social relationships in general (Chen and Feeley 2014), and the perception of being a burden (Del Sequeros et al. 2021). Differences in the perception of support and strain given to others may underlie variability in behavioral responses that lead to effective re‐engagement with social contacts versus those that avoid further social engagement and perpetuate the cycle of loneliness. These perceptions may modulate expectations about an individual's own capacity to engage in meaningful and adaptive social interactions or change expectations of how other individuals will value social contributions. Together, this evidence suggests that loneliness shapes an individual's self‐evaluation of their own participation in social relationships, which likely contributes to whether they are able to alleviate feelings of loneliness.

Further, our research expanded this work on self‐evaluation of social relationships to investigate connections with the PNS. We observed an interaction between resting HF‐HRV and loneliness predicting perceived strain given to family; however, this association was reduced to a trend after adjusting for relevant covariates. This pattern suggested that loneliness was more strongly associated with perceived strain for individuals with lower resting HF‐HRV. For individuals with higher resting HF‐HRV, this relationship was attenuated, suggesting a difference in lonely individuals' perceptions may be linked to resting PNS activity. Resting HF‐HRV reflects activation in central regulatory areas such as the prefrontal cortex that play a role in complex social and emotional responses (Balzarotti et al. 2017; Smith et al. 2017). Higher resting HF‐HRV may therefore index individual capacity to respond to the stress of loneliness with effective, goal‐directed behaviors needed for successful social engagement. On the other hand, lower HF‐HRV at rest is indicative of decreased inhibitory outflow to regulate reflexive threat reactions. This pattern may suggest that lonely individuals with higher resting HF‐HRV have a greater flexibility in the ability to regulate emotions and behavior, allowing them to manage loneliness‐related shifts in the perception of social information more adaptively. A greater capacity to use negative self‐evaluations may allow lonely individuals to prevent or remedy strain in relationships, resulting in less strain and facilitating more effective social connection. Notably, prior work has found a similar pattern, showing that resting HF‐HRV moderates individual differences in loneliness‐related behaviors, with higher resting HF‐HRV predicting greater use of approach behaviors in lonely individuals (Smith and Pollak 2022). Alternatively, individuals with higher resting HF‐HRV may be less likely to have negative self‐perceptions in the first place.

Interestingly, resting HF‐HRV did not interact with loneliness to predict perceived strain given to friends, suggesting a specificity to the type of relationship. This sort of specificity is consistent with evidence from Shiovitz‐Ezra and Leitsch (2010) suggesting that strain from family may be more related to loneliness than strain from friends. This difference may be the result of key differences between friend and family relationships. Research on friendships suggests that these relationships tend to be formed voluntarily, are based on mutual interests, and are formed between peers of the same age (Chatters et al. 2018; Walen and Lachman 2000). In contrast, family relationships are more likely to involve a range of ages, are ascribed rather than chosen, and involve more of a sense of duty and obligation (Chatters et al. 2018; Solano 1986). Consequently, the expectation of support and strain to be provided within each of these types of relationships likely differs. We might therefore expect a stronger association with the PNS in the context of strain from family relationships due to greater variability in types of family relationships and dynamics resulting from these distinctions. We also did not find an interaction between loneliness and resting HF‐HRV in predicting support given to either friends or family. A potential explanation for this finding is that the perception of strain is a cue of possible threat within relationships, which may result in greater cognitive weight being given to this perception, as opposed to the perception of support, and therefore require greater PNS involvement for the management of this threat risk. Future work may more thoroughly examine the relationship dynamics through which loneliness is linked to perceptions of strain and lower HF‐HRV at rest to identify causal mechanisms.

The current study has several strengths, including the use of a large and diverse sample from the Midlife Development in the United States (MIDUS) series and the inclusion of measures of both support and strain given in family and friend relationships to understand variability in self‐evaluations linked to loneliness and the cardiac PNS. However, there are also limitations. We were unable to distinguish between chronically lonely individuals and those experiencing more transient loneliness. Current loneliness frameworks suggest that the temporal dynamics of loneliness are a critical component of understanding how perception is shifted (Cacioppo and Hawkley 2009; Qualter et al. 2015; Spithoven et al. 2017). Chronic loneliness has been linked to persistent alterations in stress physiology and social perception, whereas transient loneliness may reflect more dynamic social experiences. It is possible that individuals with higher resting HF‐HRV experiencing transient loneliness may be better able to flexibly adjust their behavior and reduce strain in family relationships, whereas those experiencing chronic loneliness have difficulty adapting their social responses, potentially reinforcing negative relationship patterns. Temporal dynamics of the relationships may also be an important factor impacting variation in perception of how support and strain are given in different types of relationships. For example, self‐evaluations of strain could be greater in longer relationships. The cross‐sectional nature of our data prevents us from determining the directionality of these associations. It is possible that perceived strain in relationships contributes to feelings of loneliness rather than loneliness shaping perceptions of strain. Future longitudinal studies could provide a more comprehensive understanding of the temporal relationships between support, strain, loneliness, and resting HF‐HRV. Additionally, while the internal consistency of the support and strain scales was moderate and consistent with prior work, further research is needed to determine how this variability may influence observed associations.

This study also focused on perceived support and strain in family and friend relationships, but other important sources of social support, such as romantic partners, colleagues, or even online relationships, may additionally play a role. Notably, the measures of support and strain given to family explicitly excluded provisions to spouses/partners. Perceptions of support and strain within these types of relationships may be different due to dynamics involving increased reciprocity or emotional intimacy. The MIDUS sample also consists of older adults, whose relationships are shaped by aging‐related shifts, including increased caregiving responsibilities and intergenerational ties. While they see friends less often and have fewer close confidants, older adults tend to be more satisfied with their existing friendships (Nicolaisen and Thorsen 2016). Cultural factors may also play a role in shaping the relationship between loneliness, strain, and cardiac PNS activity. Attitudes toward family obligations vary across cultures, influencing how strain is perceived and managed (Fuligni et al. 1999). Examining the differential effects of support and strain from various sources could enhance our understanding of the role of all types of social relationships in mitigating loneliness.

Our findings support the idea that loneliness is related to negative self‐evaluation of support and strain given in relationships and self‐perceived contributions in the context of autonomic functioning, though more work is needed to determine the robustness of such associations. Individuals experiencing loneliness perceive their own contributions as less valuable and more likely to create further tension in relationships. This distortion in self‐perception could create a self‐reinforcing cycle, where individuals, due to feelings of loneliness, may struggle to engage in behaviors that could strengthen social connections. There is a need for more work investigating the multifaceted effects of loneliness on affective and cognitive processing, delving into the intricate interplay between perceived support, strain from others, and self‐perceived contributions with parasympathetic functioning. Future research should particularly focus on determining the bidirectional relationship between individuals' perceptions of the support and strain received from others with how they perceive their own contributions to social relationships, and the ways in which variability in these perceptions shape the resolution or further perpetuation of loneliness. Understanding these dynamics is crucial for developing effective strategies that address the specific challenges faced by lonely individuals. By gaining deeper insights into the mechanisms underlying loneliness, researchers and practitioners can design and implement interventions that not only alleviate the immediate feelings of loneliness but also foster sustainable improvements in social connection and well‐being.

## Author Contributions

**Emily M. Kent:** conceptualization, methodology, software, formal analysis, data curation, visualization, writing – original draft, writing – review and editing. **Anita Restrepo:** methodology, writing – review and editing, visualization. **Kelly E. Faig:** methodology, writing – review and editing. **Sabina Raja:** writing – review and editing. **Stephanie J. Dimitroff:** writing – review and editing. **Karen E. Smith:** methodology, writing – review and editing. **Greg J. Norman:** writing – original draft, writing – review and editing, conceptualization, resources, supervision.

## Conflicts of Interest

The authors declare no conflicts of interest.

## Supporting information


Appendix S1.

## Data Availability

The data that support the findings of this study are openly available in Interuniversity Consortium for Political and Social Research at https://www.icpsr.umich.edu/web/ICPSR/studies/36901.
