# Supplementary material for: Loneliness Is Associated With Decreased Support and Increased Strain Given in Social Relationships
Source: Psychophysiology. 2025 Jul 10;62(7):e70105. doi: 10.1111/psyp.70105 (PMC12242097; doi:10.1111/psyp.70105)
Supplement: Supplementary file 1 — Appendix S1. [file PSYP-62-e70105-s001.docx]

**Supplemental Materials**

**Interactions with Resting RMSSD**

Consistent with results using HF-HRV, there was evidence in support of our hypothesis that resting RMSSD would moderate self-evaluations, but only for perceptions of strain. Resting RMSSD interacted with loneliness to predict strain given to family (β = -0.07, SE = 0.04, p = 0.047, 95% CI [-0.15, -0.0009]), such that the effect of loneliness on strain was most pronounced for individuals with lower resting RMSSD. A simple slopes analysis at one standard deviation above and below the mean for resting RMSSD was conducted to probe the interaction effect. At higher RMSSD levels (one SD above the mean), loneliness was significantly associated with perception of strain given to family (β = 0.12, SE = 0.05, p = 0.020, 95% CI [0.02, 0.22]). At lower resting RMSSD levels (one SD below the mean), the relationship between loneliness and strain given to family was also significant and had a steeper slope (β = 0.27, SE = 0.05, p < 0.001, 95% CI [0.17, 0.38]). This result suggests that the relationship between loneliness and strain given to family is more pronounced for individuals with lower resting RMSSD. Resting RMSSD did not interact with loneliness to predict strain given to friends (β = 0.005, SE = 0.04, p = 0.898, 95% CI [-0.69, 0.79), support given to friends (β = 0.06, SE = 0.04, p = 0.078, 95% CI [-0.007, 0.14]), or support given to family (β = 0.05, SE = 0.04, p = 0.174, 95% CI [-0.02, 0.12]).

**Interaction with Gender**

To explore the potential moderation effects of gender on the interaction between HF-HRV and loneliness, we conducted an exploratory analysis. In a model with a three-way interaction between, gender, loneliness, and HF-HRV there was no significant interaction effect associated with support given to friends (β = 0.04, SE = 0.07, p = 0.54, 95% CI [-0.10, 0.19]), strain given to friends (β = 0.03, SE = 0.08, p = 0.67, 95% CI [-0.12, 0.18]), or strain given to family (β = 0.003, SE = 0.08, p = 0.97, 95% CI [-0.15, 0.15]). However, there was a significant three-way interaction for the model predicting support given to family (β = 0.22, SE = 0.07, p = 0.003, 95% CI [0.08, 0.37]). To probe the significant three-way interaction among loneliness, baseline HF-HRV, and gender, we examined the simple slopes of loneliness predicting support given at ±1 SD of HF-HRV separately by gender. Among individuals with low HF-HRV (−1 SD), loneliness significantly predicted lower support given for both men, *b* = −0.16, *SE* = 0.07, *t*(727) = −2.15, 95% CI [−0.30, −0.01], and women, *b* = −0.45, *SE* = 0.07, *t*(727) = −6.28, 95% CI [−0.60, −0.31]. At high HF-HRV (+1 SD), loneliness also predicted lower support for both men, *b* = −0.39, *SE* = 0.09, *t*(727) = −4.48, 95% CI [−0.56, −0.22], and women, *b* = −0.23, *SE* = 0.06, *t*(727) = −3.82, 95% CI [−0.35, −0.11]. Pairwise comparisons of these slopes (FDR-corrected for six tests) indicated that at low HF-HRV, the slope was significantly more negative for women than men, *t*(727) = 2.86, *p* = .026. The difference in slope for women between low and high HF-HRV approached significance, *t*(727) = 2.40, *p* = .050, suggesting a possible buffering effect of higher HF-HRV on the link between loneliness and support in women. All other comparisons were not statistically significant after FDR correction (all *ps* > .10).

**Table S1.** Item-Level Means for Support and Strain

| Variable | Question Text | Item |
| --- | --- | --- |
| **Family Support** | | |
| Mean = 1.25 (SD = 0.57), Range = [1, 4] | How much can your family rely on you for help if they have a serious problem? | Family Support 1 |
| Mean = 1.31 (SD = 0.6), Range = [1, 4] | How much can your family open up to you if they need to talk about their worries? | Family Support 2 |
| **Friend Support** | | |
| Mean = 1.25 (SD = 0.48), Range = [1, 4] | How much do you really care about your friends? | Friend Support 1 |
| Mean = 1.69 (SD = 0.6), Range = [1, 4] | How much do you understand the way your friends feel about things? | Friend Support 2 |
| Mean = 1.29 (SD = 0.57), Range = [1, 4] | How much can your friends rely on you for help if they have a serious problem? | Friend Support 3 |
| Mean = 1.26 (SD = 0.52), Range = [1, 4] | How much can your friends open up to you if they need to talk about their worries? | Friend Support 4 |
| **Family Strain** | | |
| Mean = 3.39 (SD = 0.76), Range = [1, 4] | “How often do you make too many demands on members of your family? | Family Strain 1 |
| Mean = 2.98 (SD = 0.73), Range = [1, 4] | “How often do you criticize your family? | Family Strain 2 |
| Mean = 3.56 (SD = 0.67), Range = [1, 4] | How often do you let your family down when they are counting on you? | Family Strain 3 |
| Mean = 3.02 (SD = 0.73), Range = [1, 4] | “How often do you get on your family’s nerves? | Family Strain 4 |
| **Friend Strain** | | |
| Mean = 3.58 (SD = 0.63), Range = [1, 4] | “How often do you make too many demands on your friends? | Friend Strain 1 |
| Mean = 3.29 (SD = 0.66), Range = [1, 4] | How often do you criticize your friends? | Friend Strain 2 |
| Mean = 3.61 (SD = 0.6), Range = [1, 4] | How often do you let your friends down when they are counting on you? | Friend Strain 3 |
| Mean = 3.2 (SD = 0.68), Range = [1, 4] | How often do you get on your friends’ nerves? | Friend Strain 4 |

**Table S2** - Models adjusted for smoking status. Smoking status included as a binary variable based on participant self-report of regular smoking habits (smoke regularly v. do not smoke regularly). Standardized beta coefficients (β) and standard errors (SE) are reported. All *p*-values are corrected for false discovery rate (FDR). *p < .05; **p < .01.

|  | Model 1: Support Given to Friends  β (SE) | Model 2: Support Given to Family  β (SE) | Model 3: Strain Given to Friends  β (SE) | Model 4: Strain Given to Family  β (SE) |
| --- | --- | --- | --- | --- |
|  | | | | |
| Loneliness | -0.417^**^ | -0.238^**^ | 0.209^**^ | 0.142 |
|  | (0.060) | (0.060) | (0.066) | (0.065) |
|  |  |  |  |  |
| Baseline HF-HRV | 0.082 | 0.018 | 0.021 | 0.094 |
|  | (0.060) | (0.059) | (0.065) | (0.064) |
|  |  |  |  |  |
| Smoking Status | -0.005 | -0.111 | 0.251 | 0.331^*^ |
|  | (0.135) | (0.134) | (0.147) | (0.145) |
|  |  |  |  |  |
| Loneliness:Baseline HF-HRV | 0.063 | 0.139 | -0.047 | -0.076 |
|  | (0.059) | (0.058) | (0.064) | (0.063) |
|  |  |  |  |  |
| Constant | 0.011 | -0.008 | -0.016 | -0.056 |
|  | (0.071) | (0.071) | (0.078) | (0.077) |
|  |  |  |  |  |
|  | | | | |
| Observations | 268 | 268 | 268 | 268 |
| R^2^ | 0.158 | 0.078 | 0.058 | 0.063 |
| Adjusted R^2^ | 0.145 | 0.064 | 0.044 | 0.049 |
| Residual Std. Error (df = 263) | 0.970 | 0.965 | 1.060 | 1.044 |
| F Statistic (df = 4; 263) | 12.309^***^ | 5.539^***^ | 4.041^***^ | 4.439^***^ |
|  | | | | |
| *Note:* | ^*^p<0.05; ^**^p<0.01 | | | |

**Table S3** - Models adjusted for covariates in line with Knight et al. (2020), as well as smoking status, household income, depression (CESD), anxiety (STAI), and perceived stress (PSS). Standardized beta coefficients (β) and standard errors (SE) are reported. All *p*-values are corrected for false discovery rate (FDR). *p < .05; **p < .01.

|  | | |  |  |  |
| --- | --- | --- | --- | --- | --- |
|  |  | | | | |
|  |  |  |  |  |  |
|  | Model 1: Support Given to Friends  β (SE) | Model 2: Support Given to Family  β (SE) | | Model 3: Strain Given to Friends  β (SE) | Model 4: Strain Given to Family  β (SE) |
|  | | | | | |
| Loneliness | -0.465^***^ | -0.196 | | 0.039 | 0.039 |
|  | (0.079) | (0.087) | | (0.078) | (0.082) |
|  |  |  | |  |  |
| Baseline HF-HRV | -0.043 | -0.015 | | 0.038 | 0.060 |
|  | (0.068) | (0.075) | | (0.067) | (0.071) |
|  |  |  | |  |  |
| Smoking Status | -0.153 | -0.182 | | -0.022 | 0.098 |
|  | (0.147) | (0.162) | | (0.145) | (0.153) |
|  |  |  | |  |  |
| Gender | 0.435^**^ | 0.154 | | -0.008 | 0.125 |
|  | (0.129) | (0.142) | | (0.127) | (0.134) |
|  |  |  | |  |  |
| Age | 0.047 | 0.061 | | 0.042 | 0.028 |
|  | (0.079) | (0.087) | | (0.078) | (0.082) |
|  |  |  | |  |  |
| Medical Conditions | -0.001 | 0.131 | | 0.202 | 0.106 |
|  | (0.151) | (0.166) | | (0.149) | (0.156) |
|  |  |  | |  |  |
| Medications | -0.008 | -0.063 | | -0.182^*^ | -0.083 |
|  | (0.070) | (0.077) | | (0.069) | (0.072) |
|  |  |  | |  |  |
| Household Income | -0.202^**^ | 0.008 | | 0.119 | 0.070 |
|  | (0.075) | (0.082) | | (0.074) | (0.077) |
|  |  |  | |  |  |
| Perceived Stress | 0.032 | 0.006 | | -0.106 | -0.006 |
|  | (0.106) | (0.116) | | (0.104) | (0.110) |
|  |  |  | |  |  |
| Depression | 0.097 | -0.212 | | 0.086 | -0.112 |
|  | (0.101) | (0.111) | | (0.100) | (0.105) |
|  |  |  | |  |  |
| State-Trait Anxiety | -0.079 | 0.088 | | 0.184 | 0.285 |
|  | (0.110) | (0.120) | | (0.108) | (0.113) |
|  |  |  | |  |  |
| Loneliness: Baseline HF-HRV | 0.005 | 0.136 | | -0.022 | -0.071 |
|  | (0.066) | (0.072) | | (0.065) | (0.068) |
|  |  |  | |  |  |
| Constant | -0.294^**^ | -0.214 | | -0.112 | -0.212 |
|  | (0.141) | (0.155) | | (0.139) | (0.146) |
|  |  |  | |  |  |
|  | | | | | |
| Observations | 220 | 220 | | 220 | 220 |
| R^2^ | 0.284 | 0.124 | | 0.100 | 0.101 |
| Adjusted R^2^ | 0.243 | 0.073 | | 0.048 | 0.049 |
| Residual Std. Error (df = 207) | 0.890 | 0.978 | | 0.875 | 0.921 |
| F Statistic (df = 12; 207) | 6.853^***^ | 2.448^***^ | | 1.913^**^ | 1.938^**^ |
|  | | | | | |
| *Note:* | ^*^p<0.05; ^**^p<0.01 | | | | |
